# Frequency of molar pregnancies in a tertiary care hospital: A review of pathology department records

**DOI:** 10.12669/pjms.42.1.12437

**Published:** 2026-01

**Authors:** Bushra Sikandar, Ayesha Rasheed, Mutiba Aslam, Areeba Ashfaq

**Affiliations:** 1 Bushra Sikandar, MBBS, M.Phil., DipRCpath, CHPE Assistant Professor, Department of Pathology, Dow Medical College, Dow University of Health Sciences, Karachi, Pakistan; 2 Ayesha Rasheed, Final Year MBBS Student, Dow Medical College, Dow University of Health Sciences, Karachi, Pakistan; 3 Mutiba Aslam, Final Year MBBS Student, Dow Medical College, Dow University of Health Sciences, Karachi, Pakistan; 4 Areeba Ashfaq, Final Year MBBS Student, Dow Medical College, Dow University of Health Sciences, Karachi, Pakistan

**Keywords:** Care Hospitals, Demographic, Frequency, Molar Pregnancies, Pakistan, Retrospective

## Abstract

**Objective:**

The aim of the study was to establish the prevalence of molar pregnancies in one of the tertiary care hospitals in Karachi, Pakistan, and to evaluate the histopathology variables and maternal age.

**Methodology:**

This observational retrospective study was carried out in the Department of Histopathology, Dow Medical College (2019-2024), on 385 biopsy records. The inclusion criteria included full/partial hydatidiform moles and retained products of conception (RPOC). The SPSS v25 was used to analyze data of maternal age, type of biopsy, and histopathology with descriptive statistics.

**Results:**

Out of 385 reviewed cases, the molar pregnancies were found in 28.3% of the reviewed cases, and complete moles (40.06) were the most common. Preponderance was on younger women (16-30 years, 74.8%), RPOC (56.9) was the most common type of biopsy. Rare were invasive moles (0.32) and choriocarcinoma (0.63). Histopathology showed that there were vesicular villi (30.31%) and trophoblastic proliferation (25.45%), which meet diagnostic criterion.

**Conclusions:**

The frequency of molar pregnancies and especially complete moles is very high which implies the necessity of a higher level of diagnostic accuracy and local epidemiological surveillance.Future investigations using diverse centers (e.g., DNA genotyping) should be conducted in order to narrow-down molar pregnancy sub-classification.

## INTRODUCTION

Gestational trophoblastic disease (GTD) (also known as molar pregnancy) is a rare pregnancy complication, which is an abnormal growth of trophoblastic tissue.^1^ It includes both complete and partial hydatidiform moles which have different genetic etiologies and clinical manifestations with incidences of molar pregnancies being higher in Asia (1-2 per 1000 pregnancies) in comparison with the Western countries (0.6-1.1 per 1000 pregnancies).^2^,^3^

Although molar pregnancy is a rare condition, it still has serious clinical consequences, such as the possibility of malignant transformation into choriocarcinoma, which requires strict follow-ups.^4^ Although the frequency of molar pregnancy has been studied locally in Pakistan, the data on large tertiary care centers in Karachi is scarce. As an example, a study by Lahore showed frequency of 2.9%, and a study in Quetta indicated one in 384 pregnancy incidence.^5^,^6^ But epidemiological evidence can demonstrate regional differences in incidence and presentation, which is important in maximizing the available healthcare resource allocation, pay awareness efforts to the at-risk groups, as well as to adhere to standardized diagnostic and follow-up protocols in the current clinical model.^7^

The objective of this study was to fill this gap by examining the prevalence of molar pregnancy at Dow Medical College (DMC), Karachi among six years (2019-2024). Through examining the pathology records, this research would help in the understanding of epidemiology of GTD in Pakistan, establish trends, and work towards the strategies towards health of the people.

## METHODOLOGY

The design used in this study was a retrospective design, which involves observation, to examine the prevalence of molar pregnancy using existing histopathology log data. The study by looking into archived biopsy records prevents direct contact with patients hence complies with the ethical standards of retrospective research. The study was conducted in the Department of Histopathology, Dow Medical College (DMC), Dow University of Health Sciences (DUHS), Karachi. DMC, being a large tertiary care facility, has a large case load, with approximately 3000 to 4000 cases of gynecological surgical specimen each year, and this data is extensive enough to effectively assess the prevalence of molar pregnancies in this area. Biopsy records of the study covered in the period between January 2019 and December 2024, which ensured that there is a six-year dataset of the record to follow a trend.

### Ethical Approval:

The collection of data took place over six months after the approval of the Institutional Review Board (IRB) through reference number IRB-3786/DUHS/EXEMPTION/2024/17; dated January 15, 2025.

### Eligibility criteria:

The research encompassed all biopsy specimens in women with molar pregnancy, complete and partial hydatidiform moles and retained products of conception (RPOC) that contained adequate tissues, to analyze. Cases were excluded in case only the blood clots were found in the specimen or in case it did not contain enough material because this could not be a conclusive source of diagnostic information. This being a retrospective study, all the eligible cases in the given six years-time frame were considered, thus, using a census sampling methodology. The pathology archives were searched systematically to obtain all the eligible cases over the specified time.

### Outcome measures:

Data were collected manually on the histopathology registers and biopsy request forms of Pathology department at DMC. Major variables measured comprised of the frequency of molar pregnancies, maternal age, histopathological subtype (complete or partial mole) and the year of diagnosis. The study kept the patient confidentiality by anonymizing all the data and no personal identifiers were left in the study records.

### Quality control measures:

To verify accuracy of the data, the register entries were compared to the biopsy forms to ensure that there was consistency in the data. The data extraction was considered under supervision to reduce the amount of errors, and it was also reviewed by the research team periodically to increase the reliability. This methodology complies with the IRB requirements in terms of retrospective studies and the validity of the study results.

### Statistical analysis:

Statistical Analysis of the data collected was conducted on IBM Statistical Package of social sciences (SPSS) Software (Version 25). The molar pregnancy prevalence was summarized using descriptive statistics (frequencies and percentages). The cross tabulation and the 2*2 contingency table were used to categorize the distribution of the subtypes in regards to different years. Also, prevalence estimates were made with the 95% confidence intervals so that the statistical reliability could be achieved.

## RESULTS

The retrospective review of the medical records analyzed 385 cases, and the average age was 28.16+-5.838 years (16-45 years). Most of the patients (74.8%) were aged between 16-30 years. In terms of type of biopsy, retained products of conception (RPOC) was 56.9%, and the next one was molar tissue/pregnancy (28.3%). Suction & Evacuation occupied the 11.9% of cases with Manual Vacuum Aspiration (MVA) and Dilation and Evacuation (D&E) being less common (1.6% and 1.3% respectively) (Table-I).

**Table-I:** Distribution of age groups and biopsy types in cases of suspected molar pregnancy (n=385).

Variables	Categories	Frequency (n)	Percentage (%)
Age	16-30 years	288	74.8
	31-45 years	97	25.2
Biopsy Type	RPOC	219	56.9
	Suction & Evacuation	46	11.9
	D & E	5	1.3
	MVA	6	1.6
	Molar Tissue/Pregnancy	109	28.3

Retained products of conception (RPOC); Manual vacuum aspiration (MVA); Dilation and evacuation (D&E).

The cross-tabulation compares the cases (n=385) based on the age and the type of biopsy, and it indicates that there are different trends in the process of diagnosis of a possible molar pregnancy. In patients aged 16-30 years (n=288, 74.8%), retained products of conception (RPOC) was the most frequent type of biopsy (158 cases, 55.9% of the age group), and molar tissue/pregnancy (85 cases) was the second most frequent type of biopsy. However, the 31-45 age group (n=97, 25.2) had comparatively less RPPC (61 cases, 62.9% of the age group) and molar pregnancy (24 cases, 24.7) specimen (Table-II).

**Table-II:** Age distribution across different uterine evacuation procedures and biopsy types in suspected molar pregnancy cases (n=385).

		Biopsy Type					Total
		RPOC	Suction & Evacuation	D & E	MVA	Molar Tissue/ Pregnancy	
Age	16-30 years	158	36	4	5	85	288
	31-45 years	61	10	1	1	24	97
Total		219	46	5	6	109	385

Retained products of conception (RPOC); Manual vacuum aspiration (MVA); Dilation and evacuation (D&E).

Pregnancy-related conditions had the greatest diagnosis (46.06%), and the most common one was complete molar pregnancies (40.06%). Miscarriages constituted 18.30% of the cases with the most prevalent subtype being incomplete miscarriage (11.67%). Abortions reflected 12.30% of cases mainly incomplete (6.31%) (Table-III).

**Table-III:** Frequency distribution of pregnancy-related pathologies in a clinical cohort (n=317).

Variables	Categories	Frequency (n)	Percentage (%)
Abortion (n=39)	Complete	1	0.32
	Incomplete	20	6.31
	Induced Septic	1	0.32
	Inevitable	1	0.32
	Missed	16	5.05
Miscarriage (n=58)	Incomplete	37	11.67
	Missed	17	5.36
	Complete	3	0.95
	Recurrent	1	0.32
Gestational Trophoblastic Disease (n=135)	Complete Hydatidiform Mole	127	40.06
	Partial Hydatidiform Mole	2	0.63
	Suspected Molar Pregnancy	14	4.42
	Invasive Mole	1	0.32
	Choriocarcinoma	2	0.63
	Residual Molar Tissue	1	0.32
Other pregnancy related condition (n=65)	Retained Products of Conception (RPOC)	39	12.30
	Postpartum Hemorrhage (PPH)	16	5.05
	Puerperal Sepsis	6	1.89
	Molar Tissue (Unspecified)	2	0.63
	Ectopic Pregnancy	2	0.63

**Fig.1:**
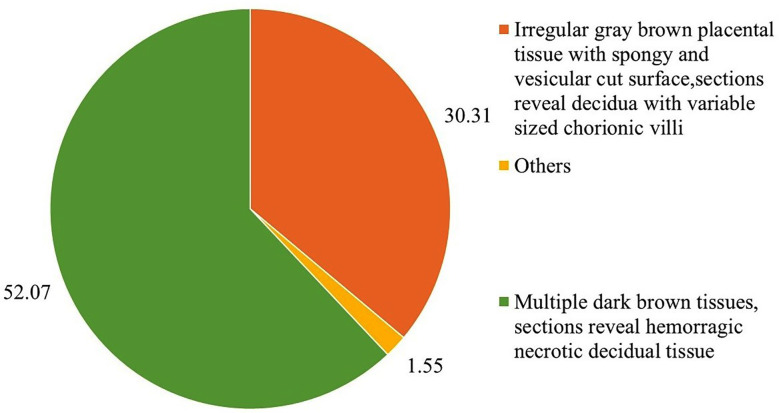
Histopathological features of molar pregnancy in placental tissue (n=324). Others included irregular gray brown placental tissue with spongy and vesicular cut surface, where sections revealed decidua with variable-sized chorionic villi in which circumferential trophoblastic proliferation or fetal parts on tissues from the uterus were seen.

After histopathological examination, 117 cases were identified to have irregular gray-brown placental tissue in the form of spongy and vesicular cut surface, which is typical of molar pregnancy (30.31%). These results are consistent with the abnormal trophoblastic proliferation observed in gestational trophoblastic disease, and this points to the role of histopathology in confirming molar pregnancy. The rest were non-specific such as hemorrhagic necrotic decidua (Fig.1).

## DISCUSSION

The results of this study found that molar pregnancies were frequent (28.3%) in the biopsy specimens and complete molar pregnancies (40.06%) prevailed in the case of the molar pregnancies. These high prevalence rates would be expected in concord with regional reports of South Asia, where studies from India found molar pregnancy incidence of 25.7% of abnormal pregnancy cases and research of Bangladesh found complete molar pregnancy incidence of 38.2% of cases of gestational trophoblastic disease.^8^-^11^ This geographical disparity is often attributed to differences in dietary factors (e.g., deficiency of carotene and animal fat), socioeconomic status, and possibly genetic predisposition.^12^

The data have identified youth and RPOC samples as the leading patients and samples in this group, and molar pregnancies as over twenty percent of the recorded cases, and demonstrated that proper histopathological screening is important in this group. The fact that the current study cohort of patients represented by the younger age group (16-30 years, 74.8%) was an interesting contrast to the existing literature. The current study finding is more in line with current studies in developing countries although the advanced maternal age (>35 years) is also a well-documented risk factor.^1^ A similar pattern was reported in a Nigerian research done by Onyekwelu et al. (2024), where 68.4% of molar pregnancies were reported to occur in women less than 30 years of age, which would imply that the region may have different proportions of risk factors.^13^ A higher fertility rate in younger populations, or possibly a variation in nutritional status, could be the cause of this demographic pattern, with folate deficiency, which is more prevalent in developing countries, having been identified as a risk factor in the pathogenesis of molar pregnancy.^12^

The most frequent type of biopsy is the high percentage of retained products of conception (RPOC, 56.9%) that elicits some critical clinical implications. This conclusion can be corroborated by a recent study, which reported that initial misdiagnosis of a complete abortion was observed in a few molar pregnancies in the first trimester of pregnancy due to the use of ultrasound in the diagnosis.^14^,^15^ The clinical presentation and management styles differ depending on age do not only support this conclusion. The findings of the present study highlight the importance of the histopathological analysis of all pregnancy tissues even today when resources are limited and a specialized imaging might not be a stable practice.

The occurrence of invasive moles at low rate (0.32%) and choriocarcinoma (0.63%) in the current study was of significant difference to those reported by other tertiary centers. While Beltrão et al. (2022) reported 2.1% malignant transformation in Brazil, current findings are more consistent with data from Egypt that showed only 0.8% progression to malignancy.^2^,^16^ This variation might reflect differences in diagnostic protocols. For instance, the availability and routine use of serum β-hCG monitoring and advanced imaging like Doppler ultrasound can lead to earlier detection and intervention before progression to malignancy. Alternatively, genetic or environmental protective factors in current study population might contribute to lower malignant transformation rates, a hypothesis supported by the work of Singh et al. (2021) on ethnic variations in trophoblastic disease behavior in South Asian population.^17^

The striking underrepresentation of partial moles (0.63%) compared to complete moles (40.06%) warrants particular attention. While Joyce et al. (2022) reported a near-equal distribution in their series from Ireland.^7^ This finding of the current study is more aligned to that of the northern parts of Pakistan that reported only 5% partial moles.^5^ This could be due to various reasons first, there could be a diagnostic issue of differentiating between early partial moles and hydropic abortions where specific ancillary methods, such as the p57 immunohistochemistry, could be important.^18^ Second, there could be a difference in causative factors of complete moles.^13^ Third, there could be a difference between how the samples were collected that may not reveal the subtle features of partial moles.

Findings of the current study on the histopathological changes of the vesicular villi and the trophoblastic proliferation are supportive of the International Federation of Gynecology and Obstetrics (FIGO) diagnostic criteria.^4^ However, with the discovery of hemorrhagic necrotic decidua in 1.55% cases, it was possible to note that some cases of molar pregnancies may have different manifestations, which was expressed by the researchers of Bahutair et al. (2024) regarding the possible development of various histological changes under the influence.^19^

The results of the study need to be discussed in the framework of the modern developments in the field of molar pregnancy management. Although the data in the current studies reveal comparatively low risk malignant transformation, the 2022 European Society of Gynaecological Oncology (ESGO) guidelines highlights that even low-risk cases like this one require standardized follow-up protocols.^20^ The fact that even the low-risk cases of malignant transformation are relatively high among young women specifically suggests the need to provide them with effective contraception counselling.^21^

### Strengths:

The research has a big, well-characterized cohort (n=385) of six years, which guarantees a thorough and detailed data on the molar pregnancy frequency in a tertiary care environment. Standardized criteria of histopathology used increase the accuracy of the diagnosis whereas cross-referring of biopsy forms with register reduces errors in data. Also, the introduction of various types of biopsy (e.g., RPOC, molar tissue) is beneficial in the scope of a comprehensive picture of clinical manifestations, which is consistent with the international diagnostic guidelines.^3^

### Limitations:

The inherent limitation of the retrospective design is that it prevents causal inferences, and use of manual records might lead to documentation bias. The single-center scope restricts generalizability to other populations, particularly rural areas with differing healthcare access. Furthermore, the lack of genetic testing (e.g., p57 immunohistochemistry) might have led to underdiagnosis of partial moles, a known pitfall in histopathology-based studies.^7^

## CONCLUSION

This study highlighted a high rate of molar pregnancies (28.3) which was observed in a Pakistani tertiary care cohort with a higher number of younger women experiencing the condition. Although complete moles were most prevalent, partial moles and malignant transformations with rare occurrence had higher diagnostic issues that elicit greater clinical attention. It is implicated that better patient outcomes are achievable with better diagnostic protocols and regional epidemiological monitoring.

### Recommendations:

Future multicenter studies with the inclusion of molecular methods (e.g., DNA genotyping) are necessary to improve molar pregnancy sub-classification. The epidemiological differences might be explained through the investigation of regional risk factors (e.g., nutritional deficiencies, consanguinity). Also, the adoption of digital pathology platforms could enhance inter-institutional diagnostic reproducibility and data sharing.
